# Examining the Effects of Couples’ Real-Time Stress and Coping Processes on Interaction Quality: Language Use as a Mediator

**DOI:** 10.3389/fpsyg.2018.02598

**Published:** 2019-01-15

**Authors:** Kevin K. H. Lau, Ashley K. Randall, Nicholas D. Duran, Chun Tao

**Affiliations:** ^1^Couples Coping with Stress Laboratory, Counseling and Counseling Psychology, College of Integrative Sciences and Arts, Arizona State University, Tempe, AZ, United States; ^2^DynamiCog Laboratory, School of Social and Behavioral Sciences, Arizona State University, Glendale, AZ, United States

**Keywords:** systemic-transactional model, language use, interaction quality, real-time interaction data, stress, romantic relationships

## Abstract

Stress in romantic relationships is an all-too-common phenomenon that has detrimental effects on relationship well-being. Specifically, stress can lead to negative interactions between partners and ultimately decrease relationship functioning. The systemic-transactional model of dyadic coping posits that by effectively communicating stress and coping with one’s romantic partner, couples can mitigate the deleterious effects of stress. Specifically, partners can engage in positive dyadic coping, which may foster couples’ sense of “we-ness,” strengthen their emotional connection, and facilitate their understanding of each other’s stressful experiences. However, these associations have not yet been examined during partners’ real-time stress conversations. When assessing dyadic coping, a particular aspect of interest is partners’ language use (i.e., pronouns, emotion words, and cognition words), as it may reflect the types of support they communicate to one another. Using real-time interaction data from 41 heterosexual couples, this study examined how couples’ stress and coping processes affect perceived interaction quality following discussions of stress. Specifically, language use (i.e., pronouns, emotion words, and cognition words) was assessed as a mediator on the association between observed stress communication and perceived interaction quality. Overall, results supported our hypotheses; when one partner communicated stress, the other partner responded with language use indicative of different types of dyadic coping (i.e., more you-talk and use of emotion words, less we-talk, I-talk, and use of cognition words), which were in turn associated with interaction quality in mixed directions. Implications of these findings for romantic couples are discussed.

## Introduction

Romantic partners’ experiences of stress can be detrimental to their relationship. In particular, effects of external stress (stress originating outside the relationship, such as stress from work or friends) can spill over into the relationship and create tension between partners (Bolger et al., 1989; Ledermann et al., 2010). Moreover, higher levels of external stress have been found to be associated with lower levels of relationship satisfaction (Bodenmann, 1997; Randall and Bodenmann, 2009; Randall and Bodenmann, 2017), as well as greater levels of relationship conflict (Bahr, 1979; Lavee et al., 1987; Neff and Karney, 2004). The systemic-transactional model of dyadic coping posits that partners can mitigate the deleterious effects of stress by effectively communicating and engaging in positive dyadic coping (Bodenmann, 1995, 1997, 2005). The benefits of positive dyadic coping have been well-documented in the literature (see Falconier et al., 2015, for a meta-analytic review); however, there is a dearth of literature on understanding how couples’ stress and coping processes may unfold in real-time conversations.

Previous studies examining the associations between couples’ stress and coping mechanisms on relationship functioning have largely depended on self-report assessments, which may be unreliable as partners may provide biased responses (O’Brien et al., 1994). In addition, self-report measures may not account for the intricate communication processes that occur as partners’ conversations unfold in time (e.g., explicit messages indicating how stress is impacting the partner, implicit stress communication via facial expressions). As such, more objective ways to measure partners’ real-time communication, such as behavioral coding and linguistic analysis, are needed.

Behavioral coding may be helpful in assessing partners’ stress communication by providing a systematic and objective set of codes that are assigned to partners based on their overt behaviors (Margolin et al., 1998). The coding scheme may also account for the various ways in which partners can communicate different types of stress (e.g., general, emotion-focused, problem-focused). Moreover, partners’ language, in particular the pronouns or words (e.g., emotion- or cognition-related words) they use, may reflect engagement in dyadic coping. Given that unique patterns of language use have been associated with greater relationship functioning (e.g., Borelli et al., 2013; Rentscher et al., 2013), it is likely that when partners engage in dyadic coping, they may also use more supportive language (e.g., positive emotion words).

Existing literature illustrates positive links between dyadic coping and general relationship functioning (e.g., Bodenmann et al., 2006; Ledermann et al., 2010), but findings on the association between dyadic coping and partners’ perceptions of specific conversations they have regarding their experiences of stress remain limited. As such, it is unclear how couples’ stress and coping processes during momentary interactions may contribute to their overall relationship well-being. Bodenmann’s (2000) stress-divorce model suggests that decreases in interaction quality may help explain the negative association between external stress and relationship satisfaction. Thus, when partners are able to effectively convey stress and support to each other in their conversations, they may view their interactions as more positive, which over time could improve their relationship satisfaction; however, this has yet to be examined.

Taken together, to our knowledge, no research to date has explored couples’ real-time stress and coping processes at the conversational level, and how these interactions may contribute to partners’ perceptions of interaction quality. As such, important gaps in the well-documented and empirically supported systemic transactional model of dyadic coping (Bodenmann, 1995, 1997, 2005) remain largely unexamined. To address this gap in the literature, this study examines associations between stress communication, language use, and interaction quality in discussions about stress using real-time interaction data from heterosexual couples.

### Systemic-Transactional Model of Dyadic Coping

Stress can be conceptualized on a number of dimensions, which includes its origin (see Randall and Bodenmann, 2009 for a review). Stress that originates outside the relationship (i.e., external stress), such as work and finances, can negatively impact a partner’s perception of stress within the relationship through stress Spillover (Bolger et al., 1989; Repetti and Wood, 1997). Stress Spillover occurs when one partner experiences external stress, and the emotions associated with that stress are carried over into the relationship, impeding positive interactions and effective stress communication between the partners. Stress communication refers to how partners convey and understand each other’s stressful experiences (Bodenmann, 1995, 1997, 2005). For example, following the experience of a bad interaction with a friend, one partner may come home to express their stress to the other partner, which then draws the other partner into the coping process. Importantly, a partner may communicate their stress *non-verbally* (e.g., through signs of discomfort or frustration) or *verbally*. In this study, we focus specifically on verbal stress communication.

Verbal stress communication can be categorized into three types: (1) *general or neutral explanation of stress* (i.e., describing only the facts of a stressful situation without conveying emotion, offering or seeking advice), (2) *emotion-focused stress communication* (i.e., highlighting the emotional effect of a stressor and/or describing felt emotions), and (3) *problem-focused stress communication* (i.e., focusing on tangible solutions to stressors and/or soliciting practical advice from the partner). Emotion-focused stress communication can be further distinguished based on implicit or explicit emotions. *Implicit emotion-focused stress communication* refers to when partners discuss their stress in a vague manner, without mentioning specific emotions (e.g., “Work is making me *stressed*,” “My friends made me feel *bad*”), whereas *explicit emotion-focused stress* communication occurs when partners identify specific emotions (e.g., “I am *angry* at my parents,” “I am *scared* that I will run out of money”). Prior research has examined stress communication in the form of willingness to disclose, and results suggest that effective communication is positively associated with relationship satisfaction (e.g., Meeks et al., 1998; MacNeil and Byers, 2005; Smith et al., 2008; Montesi et al., 2011). This suggests that when partners allow themselves to be open in their conversations with each other, they may become more satisfied with their partners and in their relationships. Despite these results and the robust evidence for interpersonal communication, partners’ stress communication, as conceptualized by the systemic transactional model (Bodenmann, 2005), have not yet been explicitly examined.

Another important element to the perception of interaction quality is the response from one’s interaction partner following the stress disclosure. For instance, if one partner self-discloses an upsetting experience at work and their partner responds negatively, then the stressed partner may perceive the quality of the interaction as negative (as opposed to positive). As such, it is important to also consider how partners respond to each other’s stress communication and how it may contribute to the overall interaction quality.

Following the communication of one partner’s stress, the other partner then responds in order to help mitigate (or exacerbate, in the case of negative dyadic coping) the stressed partner’s experience (Bodenmann, 2005). Positive dyadic coping can take one of three forms: (1) *emotion-focused dyadic coping* (i.e., providing emotional and empathic support), (2) *problem-focused dyadic coping* (i.e., providing partner with new perspectives and practical solutions), and (3) *delegated dyadic coping* (i.e., taking on extra responsibilities so that the partner’s workload is lessened). In this study, we focused on the verbal exchange between partners and therefore only examined emotion- and problem-focused dyadic coping, as they may be expressed verbally, as opposed to delegated dyadic coping, which may be more likely to be exhibited via action. Partners can also engage in *negative* dyadic coping (e.g., mock or invalidate partner’s feelings, provide insincere support). Higher levels of perceived positive dyadic coping and lower levels of negative dyadic coping have been found to be positively associated with reduced stress (Ledermann et al., 2010), and greater relationship quality (Bodenmann et al., 2006, 2011).

An important aspect of dyadic coping is the language that partners use with each other, given that partner’s words may facilitate the communication of the various types of support outlined above. Specifically, the use of pronouns may be salient to examine in partners’ engagement of any form of positive dyadic coping, emotion words in emotion-focused dyadic coping, and cognition words in problem-focused dyadic coping.

### Language Use and Dyadic Coping

One central aspect of the engagement in positive dyadic coping is that it can foster couples’ sense of “we-ness” or cohesion between partners (Bodenmann, 2005). The extent to which partners view themselves as a close, intimate unit may be reflected in their use of pronouns, which represents the partner’s attentional focus and identification (Tausczik and Pennebaker, 2010). For instance, partners who view the relationship as cohesive may be more likely to highlight the interdependence by using more plural, first-person pronouns like “we” and “us.” They may also conceptualize each other’s external stress as “our” stress, an issue that they must work together in order to combat. Consistent with positive dyadic coping, it has been found that greater use of plural, personal pronouns (i.e., *we-talk*) is positively associated with relationship satisfaction (Borelli et al., 2013) and communication quality (Biesen et al., 2015).

Conversely, partners who do not view themselves as cohesive may highlight their individual identities by using more singular pronouns, such as “I” and “you.” There is evidence suggesting that the use of singular, first-person pronouns (i.e., *I-talk*) is negatively correlated with relationship quality (Slatcher et al., 2008; Rentscher et al., 2013). Further, the use of singular second-person pronouns (i.e., *you-talk*) negatively predicts interaction quality in couples (Biesen et al., 2015). Partners’ use of you-talk may communicate distance between partners, and further, indicate blame and criticism (e.g., “*You* never do the dishes”). Therefore, when partners engage in any form of positive dyadic coping, they may use fewer “I” and “you” pronouns because they communicate separation between partners, which could lead to higher perceived interaction quality.

Emotion words (e.g., happy, sad, excited, and anxious) can convey individuals’ emotional responses and level of immersion to certain experiences (Tausczik and Pennebaker, 2010). Individuals may use emotion words to describe whether they feel positively about an experience via words like “cheerful,” and “joy,” or negatively with words like “hate” and “hurt.” In addition, when an individual feels burdened by a specific stressor he/she may be more willing to verbally express feelings. The use of emotion words between romantic partners has been found to be positively associated with relationship satisfaction (Slatcher and Pennebaker, 2006) as well as relationship adjustment (Baddeley and Pennebaker, 2011).

Applied to the systemic-transactional model (Bodenmann, 1995, 1997, 2005), partners’ use of *positive emotion words* during the expression of emotion-focused dyadic coping may help partners express their feelings to one another. In the context of couples’ stress-related conversations, the supporting partner’s use of positive emotion words may provide encouragement and support for the stressed partner, which in turn may impact their partner’s perception of the interaction. Conversely, the use of *negative emotion words* has not been extensively examined in the literature. Despite the lack of literature, the use of negative emotion words could be easily be argued to yield a negative perception of the interaction.

Cognition words refer to words that illustrate the processing and interpretation of information (e.g., because, know, and think). The use of cognition words has been found to benefit individuals’ recounts of past stressful events, leading to more positive mental health outcomes (Cordova et al., 2001; Boals and Klein, 2005). The use of cognition words may reflect an attempt at deeper understanding of stressors, which could be considered as an effective coping mechanism. Applied to the systemic transactional model (Bodenmann, 1995, 1997, 2005), when partners engage in problem-focused dyadic coping, they may try to understand and make meaning out of each other’s experiences; thus, they may use more words that indicate cognitive processing. As outlined above, communication about stress and coping could have positive effects on partners’ perceptions of their interactions.

### Associations Between Stress Communication, Dyadic Coping, and Interaction Quality

Communication quality is positively associated with relationship satisfaction (Litzinger and Gordon, 2005). Further, Carroll et al. (2013) found that, in a sample of 1,117 married individuals who were employed full-time employees, the use of constructive communication mediated the association between work-family conflict and relationship satisfaction. However, to our knowledge, no research has examined partners’ perceptions of their interactions immediately following their conversations about stress.

Cross-sectional research on dyadic coping has found a robust association between perceptions of partners’ dyadic coping and perceived well-being, across cultures (Falconier et al., 2016). Despite these well-documented associations, it is still unclear how partners’ stress communication and coping processes unfold during real-time. Effective stress communication, and responses of positive dyadic coping, are thought to result in more satisfying interactions, resulting in greater relationship satisfaction over time (Bodenmann, 1995, 1997, 2005); however, examinations on such associations using real-time interaction data is lacking. This manuscript addresses this gap in the literature, which is pertinent to promoting the understanding of the partners’ momentary experiences of stress communication and coping processes during real-time interactions.

### Present Study

**FIGURE 1:**
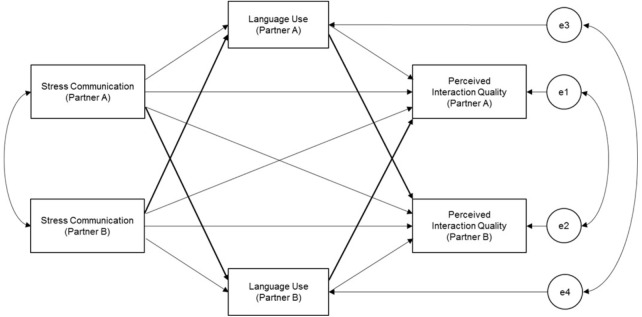
Proposed model of language use (Time 1) mediating the association between stress communication (Time 1) and interaction quality (Time 2).

The present study used real-time interaction data from 41 heterosexual couples’ discussions about external stress to examine the mediational effect of partners’ language use on the association between observed stress communication and self-reported interaction quality (Figure 1). The use of dyadic data allowed for the examination of both actor (i.e., one partner’s independent variable predicting their own outcome variable) and partner (i.e., one partner’s independent variable predicting their partner’s outcome variable) effects. Given our interest in partners’ interactions with each other during their conversations, partner effects were predicted to be more salient than actor effects, because of the transactional nature of stress communication and dyadic coping. Further, the association between stress communication and interaction quality was expected to be more pronounced for the partner who communicates their stress (i.e., assigned to discuss their external stress topic). The following hypotheses (H) were tested:

H1: It was hypothesized that the use of pronouns (e.g., we, I, you) would mediate the association between stress communication and perceived interaction quality. Specifically, when one partner engaged in *general stress communication* (i.e., neutral explanation of his/her stress), the other partner would respond with greater use of *we-talk* (H1a) and lower use of *I-talk* (H1b) and *you-talk* (H1c), which would then be positively associated with perceived quality of the interaction for the stressed partner.
H2: It was hypothesized that the use of emotion words would mediate the association between stress communication and perceived interaction quality. Specifically, when one partner engaged in *emotion-focused stress communication*, the other partner would respond with more *positive emotion words* (e.g., happy, cheer, enjoy; H2a) and fewer *negative emotion words* (e.g., sad, angry; H2b), which would then be positively associated with the first partner’s perceived quality of the interaction.
H3: It was hypothesized that the use of words related to cognitive processing (e.g., think, believe, and maybe) would mediate the association between stress communication and perceived interaction quality. Specifically, when one partner engaged in *problem-focused stress communication* (e.g., seeking practical advice or alternative perspective), the other partner would respond with more *cognition words*, which would then be positively associated with interaction quality for the first partner.

## Materials and Methods

### Participants

The initial sample for this study consisted of 54 heterosexual couples (*N* = 108 individuals) that completed the baseline questionnaire; however, only 41 couples (*n* = 82 individuals) completed both the baseline questionnaire and participated in the laboratory session; as such, results are based on the 41 couples. The mean age of women was 30.39 years (*SD* = 7.36) and the mean age of men was 30.41 years (*SD* = 6.87). The majority of participants identified as White (*n* = 58), followed by Hispanic (*n* = 14), Asian American (*n* = 4), Native Hawaiian or Pacific Islander (*n* = 2), African American (*n* = 1), and 3 individuals identified with other racial backgrounds. Most participants reported having obtained a terminal college, university, or graduate degree (*n* = 38 women, 29 men). The median range of annual income was $25,000 to $50,000.

Partners reported being together, on average, for 5.45 years (*SD* = 5.25). Out of the 41 couples, 4 reported that they were in committed relationships and were not cohabiting, 6 indicated that they were in committed relationships and cohabiting, 2 were engaged and not cohabiting, 10 were engaged and cohabiting, and 19 were married. Eleven of the couples reported having children.

### Procedure

This study’s procedures were reviewed and approved by the Institutional Review Board at Arizona State University, and all participants consented to participate. Participants were recruited via advertisements posted on Craigslist, Facebook, and electronic mailing lists belonging to various universities and professional organizations in a Southwestern region of the United States.

Data for this study were collected in three parts: (1) a screening survey to ensure couples’ eligibility, (2) an initial baseline questionnaire, and (3) a laboratory session. Couples were required to fulfill the following criteria in order to participate: (1) both partners were over the age of 18; (2) had been in a relationship with their current partner for at least 6 weeks; and (3) both partners were willing to participate in the study. Eligible participants were electronically sent the baseline questionnaires, which contained demographic measures and measures related to relationship functioning. Participants were instructed to complete the baseline questionnaires separately. The baseline questionnaire took approximately 1 h to complete.

Following the completion of the baseline questionnaire, participants were scheduled for a laboratory session wherein they were asked to engage in a series of video-taped conversations. Each couple was asked to have a conversation regarding a source of *external stress* (i.e., originating outside of the relationship), *internal stress* (i.e., coming from within the relationship), and a topic of *enjoyment*. Each video-taped conversation lasted for 6 min, and couples saw a message on a screen in the laboratory alerting them that they have 1 min left for the conversation. For the present study, only the discussions about external stress were used based on previous evidence indicating that external stress strongly predicts relationship outcomes (e.g., Story and Repetti, 2006; Bodenmann et al., 2007; Randall and Bodenmann, 2009; Randall and Bodenmann, 2017).

Topics were determined by using the Multidimensional Stress Scale for Couples (Bodenmann, 2006), which was included in the baseline questionnaire and assessed partners’ experience of various external stressors. The research team chose the external stressor that each partner rated as the most stressful. Topics were counterbalanced by partner gender (e.g., Couple 1: Female’s external stress, Couple 2: Male’s external stress, etc.). For each conversation, partners were instructed to discuss the chosen topic for 6 min. Following the conversation, partners were given a brief questionnaire asking about their perception of the interaction.

Each couples’ conversation was transcribed by a team of eight undergraduate research assistants using the Praat software (Boersma and Weenink, 2006). After all transcripts had been completed, a different team of three graduate research assistants checked them for accuracy. The video recordings of the stress discussions were separated into 10-s segments to prepare for the behavioral coding, resulting in a total of 36 segments for each 6-min conversation. A team of three graduate research assistants, all familiar with the systemic transactional model of dyadic coping, then reviewed the videos according to the codebook (Lau et al., unpublished) and indicated whether partners exhibited signs of non-verbal and verbal stress communication and dyadic coping. After initial training, the graduate research assistants were blindly assigned videos to complete coding independently, and some of the conversations were viewed by all three of them in order to calculate inter-rater reliability (see below).

### Measures

#### Observed Stress Communication

Partners’ observed stress communication was measured using the English-version of Bodenmann’s (2008) dyadic coping behavioral coding system (Lau et al., unpublished). The Dyadic Coping Manual outlines types of non-verbal and verbal stress communication and dyadic coping. For the purpose of this study, we utilized data from the verbal stress communication codes. The four types of verbal stress communication are: (1) *general or neutral explanations of stress* (e.g., “I have a work to do and my co-workers are not helping”), (2) *implicit emotion-focused stress communication* (e.g., “I *do not like* how my friend talked to me”), (3) *explicit emotion-focused stress communication* (e.g., “I am furious at my boss”), and (4) *problem-focused stress communication* (e.g., “What do you think I should do in this situation?”).

The average Cohen’s Kappa values, across all external stress conversations in this study for the three raters was 0.82, indicating high inter-rater reliability (Cohen, 1968). To maintain consistency with the LIWC measures (described below), percentages were calculated for each partner based on the number of times that each partner was observed engaging in stress communication during the conversations by dividing the number of segments in which one form of stress communication occurred by the total of 36 segments.

#### Self-Reported Interaction Quality

Participants rated their perceived quality of the external stress interaction following their conversation. Partners responded to 19 items related to relationship satisfaction and quality on a 7-point scale, ranging from 1 (*strongly disagree*) to 7 (*strongly agree*). Examples of items included, “In the previous interaction, my partner communicated warmth rather than coldness,” and, “In the previous interaction, I felt that my partner understood what I was saying.” Some items were originally rated a 7-point scale, ranging from 1 (*a negative personality trait*) to 7 (*a positive personality trait*), which were then recoded to be consistent with the scale and examples described above. For example, one item was, “In the previous interaction, my partner was 1 (*untrustworthy*) versus 7 (*trustworthy*),” and this was revised to, “In the previous interaction, my partner was trustworthy,” rated between 1 (*strongly disagree*) and 7 (*strongly agree*). A total score was generated by averaging all 19 of the items, with higher scores indicating greater levels of satisfaction with the interaction. Cronbach’s alphas for the 19 items were 0.93 for both females and males, demonstrating high internal reliability (Cronbach, 1951).

#### Language Use

To obtain data related to partners’ language use, raw transcript data were analyzed using the Linguistic Inquiry and Word Count (LIWC) computer program (Pennebaker et al., 2015). LIWC calculated percentages of all pronoun types, emotion words, and cognition words in the total word count of a given text sample. Based on prior literature (Rohrbaugh et al., 2012), the transcripts were prepared in the following ways prior to running them in LIWC: (1) raw transcripts were split by speaker and all information other than the actual speech and an identifying marker was removed; and (2) filler words and expressions that contained pronouns that did not carry independent meaning (e.g., “I” in “I mean”) were marked in a way that prevented LIWC from counting them toward this category. The resulting percentages of pronouns (e.g., “I”; “you”; “we”), emotion words (e.g., “happy,” “sad,” “scared”), and cognition words (e.g., “think,” “because,” “effect”) in total word counts were used in our current analyses.

### Analytic Plan

Dyadic data contains many sources of interdependence. Given this, prior to conducting dyadic data analysis, it is recommended to determine the distinguishability of partners’ roles (Kenny et al., 2006). Typically, research with heterosexual couples has used gender as a distinguishing variable (i.e., female and male); however, in our study, we must also consider the roles of the stress communicator and the listener. Thus, we conducted two separate sets of tests—one with gender and the other with speaker-listener roles as the distinguishable variable—to determine distinguishability. Speaker-listener roles were initially determined with the study design (selecting one partner’s stress as the topic of discussion), and we used the behavioral coding of stress communication to verify role assignments and made changes based on who communicated more stress during the real-time conversations. The tests of distinguishability involved comparing the -2 log likelihood goodness of fix indices of the hierarchical linear models with and without the distinguishable variables; if the difference between the goodness of fit is significant according to chi-square calculations, then it would indicate that the model with the distinguishable has greater fit and partners are assumed to be distinguishable based on the examined variable. The tests showed that neither the models with gender nor the ones with speaker-listener roles as the distinguishable variables had significantly better fit than the indistinguishable model, suggesting that partners’ results did not differ based on gender or speaker-listener roles. To account for the indistinguishability, data was restructured using the “double-entry method” suggested by Kenny et al. (2006), such that each partner’s scores were entered twice, once as the actor and again as the partner, and adjustments are made to the weights of the data points and the degrees of freedom.

The Actor-Partner Interdependence Mediation Model (APIMeM; Ledermann et al., 2011) was used to analyze the restructured dataset. The method of analysis has three functions: (1) accounts for variability due to the interdependence of partners, (2) assesses the impact of one partner’s predictor and mediator variables on both partners’ outcomes, and (3) measures the residual covariance between the variable pairs. In this model, there are actor and partner effects between each predictor, mediator, and outcome variable, along with direct and indirect effects from the standard mediation model, resulting in a total of 12 paths (see Figure 1).

Given our hypotheses, we present the results from the partner-partner effects (i.e., the association between one partner’s stress communication, the other partner’s language use, and the first partner’s interaction quality) for parsimony. Data analyses were conducted using Structural Equation Modeling (SEM) with Mplus 8, which is the suggested method to test the APIMeM as it estimates all model parameters within a single equation (Cook and Kenny, 2005; Ledermann et al., 2011).

## Results

### Descriptive Analyses

Means, standard deviations, and bivariate correlations among the study variables are presented in Table 1. The only significant difference between female and male partners was their engagement in problem-focused stress communication, *t*(40) = -2.44, *p* = 0.01. Specifically, male partners were observed to engage in more problem-focused stress communication, compared to their female partner. Results for the APIMeMs are described below in terms of actor and partner effects due to the indistinguishability of Partner A’s and B’s roles. All models showed good fit (Table 2).

**Table 1:** Descriptive statistics and pairwise correlations between study variables.

	Descriptive Statistics														
	Female		Male			Pairwise Correlations									
Variables	*M*	*SD*	*M*	*SD*	*t*	1	2	3	4	5	6	7	8	9	10
(1) GSC	25.00	20.85	25.54	22.41	0.09	**-0.57^∗∗^**	0.02	-0.12	0.18	0.22	-0.42^∗∗^	-0.26	-0.19	0.16	0.05
(2) EmoSC	5.28	8.54	5.22	6.09	-0.04	0.30	**-0.20**	0.23	-0.05	0.44^∗∗^	-0.25	-0.03	0.07	0.22	-0.03
(3) ProbSC	7.18	12.00	3.32	6.49	-2.44^∗^	-0.16	-0.09	**0.53^∗∗∗^**	0.13	-0.02	0.24	-0.29	-0.14	0.20	-0.01
(4) IntQ	5.57	0.99	5.73	0.87	1.05	-0.09	-0.07	-0.15	**0.45^∗∗^**	0.14	-0.38^∗^	0.10	0.04	0.17	0.11
(5) We-talk	2.00	1.60	1.90	1.70	-0.56	-0.37^∗∗^	-0.36^∗^	0.21	0.14	**0.76^∗∗∗^**	-0.04	-0.27	0.13	-0.10	-0.24
(6) I-talk	6.73	2.59	6.81	2.18	0.14	0.26	0.39^∗^	0.03	-0.06	-0.38^∗^	**-0.12**	-0.51^∗∗^	-0.22	0.24	0.20
(7) You-talk	3.68	2.67	2.87	1.71	-1.50	-0.27	-0.26	-0.12	-0.26	-0.08	-0.42^∗∗^	**-0.22**	-0.07	-0.15	-0.06
(8) PosEmoW	4.59	2.64	4.49	3.23	-0.16	-0.32^∗^	-0.31^∗^	-0.17	0.15	0.13	-0.16	0.24	**0.01**	0.39^∗^	-0.31^∗^
(9) NegEmoW	1.16	1.23	1.11	0.86	-0.33	0.30	0.57^∗∗∗^	-0.05	0.06	-0.14	0.30	-0.09	-0.26	**0.56^∗∗∗^**	0.03
(10) CogW	14.78	2.31	14.34	1.96	-0.97	0.22	-0.05	-0.30	-0.10	0.02	0.11	-0.10	-0.03	-0.04	**0.06**

*GSC, General Stress Communication; EmoSC, Emotion-focused Stress Communication; ProbSC, Problem-focused Stress Communication; IntQ, Interaction Quality; PosEmoW, Positive Emotion Words; NegEmoW, Negative Emotion Words; CogW, Cognitive Processing Words. The mean values for all variables except IntQ are reported in percentages in proportion to the entire stress conversation. IntQ is assessed on a 7-point scale, ranging from 1 (strongly disagree) to 7 (strongly agree). Females’ bivariate correlations are displayed above the main diagonal (green), the males’ are below the diagonal (in orange), and correlations between females and males are bolded on the main diagonal. ^∗^p < 0.05, ^∗∗^p < 0.01, ^∗∗∗^p < 0.001.*

**Table 2:** Fit indices for all models with interaction quality as dependent variable.

		Model fit					
IV	Mediator	χ^2^	df	*p*	CFI	RMSEA	SRMR
GSC	–	0.14	2	0.93	1.00	0.00	0.01
GSC	We-talk	5.54	6	0.48	1.00	0.00	0.06
GSC	I-talk	1.01	6	0.99	1.00	0.00	0.02
GSC	You-talk	3.01	6	0.81	1.00	0.00	0.05
EmoSC	–	0.50	2	0.78	1.00	0.00	0.03
EmoSC	PEmoW	4.02	6	0.67	1.00	0.00	0.06
EmoSC	NEmoW	1.73	6	0.94	1.00	0.00	0.03
ProbSC	–	0.48	2	0.78	1.00	0.00	0.03
ProbSC	CogW	2.70	6	0.85	1.00	0.00	0.07

*IV, Independent Variable; CFI, Comparative Fit Index; RMSEA, Root Mean Square Error of Approximation; SRMR, Standardized Root Mean Square Residual; GSC, General Stress Communication; EmoSC, Emotion-focused Stress Communication; ProbSC, Problem-focused Stress Communication; PEmoW, Positive Emotion Words; NEmoW, Negative Emotion Words; CogW, Cognition Words.*

### H1: Pronouns Mediate the Association Between General Stress Communication and Interaction Quality

#### We-Talk

**FIGURE 2:**
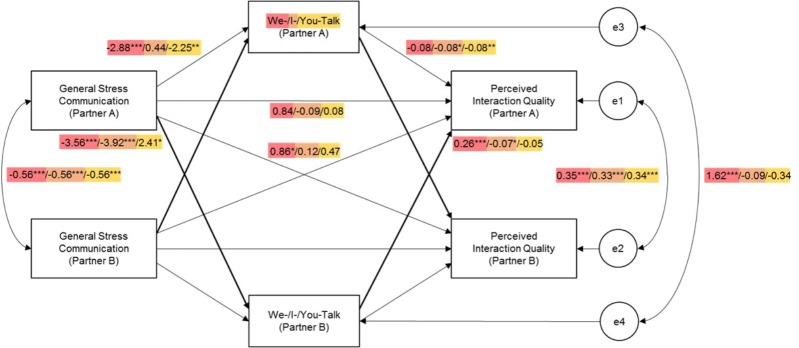
Unstandardized model results of we-talk (red), I-talk (orange), and you-talk (yellow) mediating the association between general stress communication and perceived interaction quality. Only one set of coefficients were included due to the indistinguishability of partner roles. ^∗^*p* < 0.05, ^∗∗^*p* < 0.01, ^∗∗∗^*p* < 0.001.

Results revealed a significant partner effect of *general stress communication* on the use of *we-talk* (*b* = -3.56, *SE* = 0.84, *p* < 0.001; Figure 2), such that one partner’s general stress communication was negatively associated with the other partner’s we-talk. There was also a significant partner effect of *we-talk* on *interaction quality* (*b* = 0.26, *SE* = 0.05, *p* < 0.001). The direct actor effect of Partner A’s general stress communication on his/her own interaction quality was marginally significant, *b* = 0.84, *SE* = 0.48, *p* = 0.08, and further, the partner-partner indirect effect was statistically significant, *b* = -0.93, *SE* = 0.31, *p* = 0.002. Thus, these findings suggest that we-talk partially mediated the association between general stress communication and perceived interaction quality, supporting H1a.

#### I-Talk

Results indicated a significant partner effect of *general stress communication* on *I-talk* (*b* = -3.92, *SE* = 0.99, *p* < 0.001; Figure 2), such that one partner’s general stress communication was negatively associated with the other partner’s I-talk. In addition, we found a significant partner effect of *I-talk* on *interaction quality* (*b* = -0.07, *SE* = 0.03, *p* = 0.04) as well as a significant actor effect (*b* = -0.08, *SE* = 0.03, *p* = 0.02). The direct actor effect was not significant, *b* = -0.09, *SE* = 0.47, *p* = 0.86; however, the partner-partner indirect effect was, *b* = 0.26, *SE* = 0.13, *p* = 0.05. Therefore, I-talk fully mediated the association between general stress communication and interaction quality, which provided support for H1b.

#### You-Talk

Results showed a significant partner effect of *general stress communication* on *you-talk* (*b* = 2.41, *SE* = 1.07, *p* = 0.02; Figure 2), such that one partner’s general stress communication was positively associated with the other partner’s you-talk. However, no significant partner effect was found between *you-talk* and *interaction quality* (*b* = -0.05, *SE* = 0.04, *p* = 0.15). The direct actor effect was not significant, *b* = 0.47, *SE* = 0.36, *p* = 0.20, and neither was the indirect partner-partner effect, *b* = -0.13, *SE* = 0.10, *p* = 0.21. Therefore, our hypothesis related to our focus on actor-partner-actor effects (H1c) was not supported. However, we found additional results based on the other paths that were not hypothesized. There was a significant actor effect of *general stress communication* on *you-talk* (*b* = -2.25, *SE* = 0.76, *p* = 0.003) as well as a significant actor effect of *you-talk* on *interaction quality* (*b* = -0.08, *SE* = 0.03, *p* = 0.002). While the direct actor effect was not significant, *b* = 0.08, *SE* = 0.46, *p* = 0.87, the indirect actor-actor mediational effect was, *b* = 0.19, *SE* = 0.10, *p* = 0.05. Although not hypothesized, it was found that the actor’s you-talk mediated the association between his/her own general stress communication and interaction quality.

### H2: Emotion Words Mediate the Association Between Emotion-Focused Stress Communication and Interaction Quality

#### Positive Emotion Words

**FIGURE 3:**
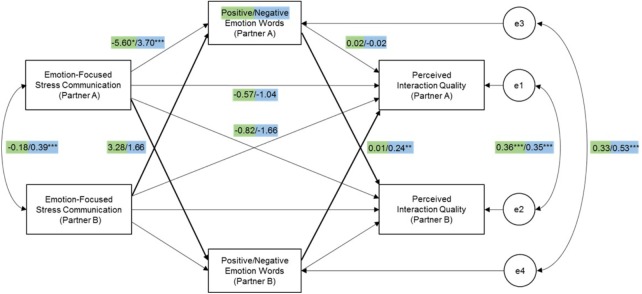
Unstandardized model results of positive (green) and negative emotion words (blue) mediating the association between emotion-focused stress communication and perceived interaction quality. Only one set of coefficients were included due to the indistinguishability of partner roles. ^∗^*p* < 0.05, ^∗∗^*p* < 0.01, ^∗∗∗^*p* < 0.001.

Results showed no significant partner effect of *emotion-focused stress communication* on the use of *positive emotion words* (*b* = 3.28, *SE* = 2.93, *p* = 0.26; Figure 3) and no significant partner effect of *positive emotion words* on *interaction quality* (*b* = 0.01, *SE* = 0.03, *p* = 0.67). The indirect partner-partner effect was not significant, *b* = 0.04, *SE* = 0.15, *p* = 0.76, and neither was the direct actor effect, *b* = -0.57, *SE* = 0.97, *p* = 0.55. The only significant association found in this model was between one partner’s *emotion-focused stress communication* with his/her own use of *positive emotion words* (*b* = -5.60, *SE* = 2.61, *p* = 0.03). Our findings do not support H2a regarding the mediation of positive emotion words on the association between emotion-focused stress communication and interaction quality.

#### Negative Emotion Words

Results showed a marginally significant partner effect of *emotion-focused stress communication* on the use of *negative emotion words* (*b* = 1.66, *SE* = 0.93, *p* = 0.07; Figure 3) and a significant partner effect of *negative emotion words* on *interaction quality* (*b* = 0.24, *SE* = 0.07, *p* < 0.001). The direct actor effect was not significant, *b* = -1.04, *SE* = 0.88, *p* = 0.24, however, the indirect partner-partner effect was marginally significant, *b* = 0.40, *SE* = 0.25, *p* = 0.10. Based on these results, there is limited support for the mediating role of negative emotion words in the association between emotion-focused stress communication and interaction quality (H2b). In addition to these main findings, it was also found that stress communication was positively associated with one’s own use of negative emotion words (*b* = 3.70, *SE* = 1.03, *p* < 0.001), and that there was a significant indirect partner-partner effect of stress communication on one’s own use of negative words on the partner’s interaction quality (*b* = 0.89, *SE* = 0.42, *p* = 0.04).

### H3: Cognitive Processing Words Mediate the Association Between Problem-Focused Stress Communication and Interaction Quality

**FIGURE 4:**
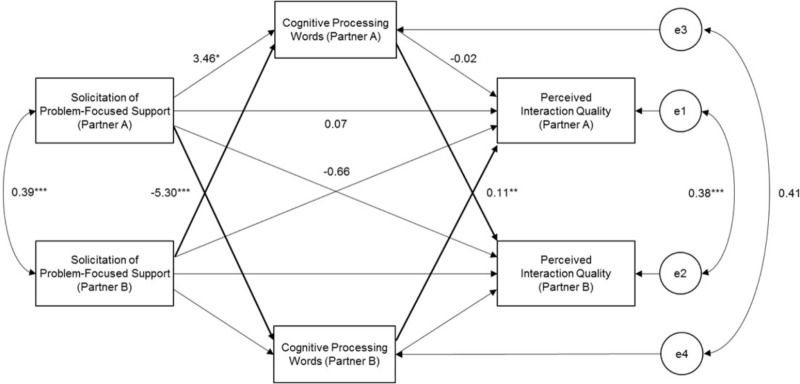
Unstandardized model results of cognitive processing words mediating the association between problem-focused stress communication and perceived interaction quality. Only one set of coefficients were included due to the indistinguishability of partner roles. ^∗^*p* < 0.05, ^∗∗^*p* < 0.01, ^∗∗∗^*p* < 0.001.

Lastly, we found a significant partner effect of *problem-focused stress communication* on *cognition words* (*b* = -5.30, *SE* = 1.15, *p* < 0.001) and a significant partner effect of *cognition words* on *interaction quality* (*b* = 0.11, *SE* = 0.03, *p* = 0.002). The direct actor effect was not significant, *b* = 0.07, *SE* = 0.85, *p* = 0.94, while indirect partner-partner effect was, *b* = -0.57, *SE* = 0.23, *p* = 0.01 (Figure 4). Overall, there is support for H3, in that cognitive processing words partially mediated the association between problem-focused stress communication and interaction quality. However, the association between stress communication and use of cognition words was negative, which is different from what we had hypothesized.

In sum, there was partial support for our hypotheses. Specifically, we-talk partially mediated and I-talk fully mediated the association between general stress communication and perceived interaction quality. Moreover, the use of cognitive processing words mediated the association between problem-focused stress communication and perceived interaction quality.

Although the focus of this study was on the partner effects of stress communication on language use (i.e., the association between one partner’s stress communication and the other partner’s language use) to highlight the interaction between the actor and the partner, there were other notable findings. For instance, results revealed significant negative actor effects between stress communication and we-talk, you-talk, and positive emotion words, such that when one communicates stress, he/she also uses fewer “we” and “you” pronouns and positive emotion words.

## Discussion

Partners’ experience of external stress is a common occurrence, which can lead to significant relational concerns and even dissolution if not properly dealt with (e.g., Kiecolt-Glaser et al., 2003; Bodenmann et al., 2007; Falconier et al., 2015). In order to promote relationship well-being, it is imperative that relationship researchers understand how partners can effectively manage stress via communication and coping, particularly when discussing stressful situations. To our knowledge, this study was the first to examine the stress and coping processes described by the systemic-transactional model at the real-time conversational level. Specifically, we tested whether partners’ observed engagement in stress communication and dyadic coping, more specifically in the form of language use, would contribute to their perceptions of quality of their real-time interactions about external stressors.

### General Stress Communication, Pronouns, and Interaction Quality

Overall, the results supported our hypotheses regarding the mediator role of pronoun use. First, the results indicated that we-talk is a significant mediator in the association between stress communication and interaction quality. However, contrary to the hypothesis, the association between stress communication and we-talk was negative. That is, when one partner communicated their stress in a general or neutral manner, the other partner responded with less we-talk, which then positively predicted interaction quality. We-talk has been typically thought by researchers to communicate cohesion between couples and in fact, found to be associated with relationship satisfaction (Borelli et al., 2013). It is possible that when the supporting partner used fewer “we” words, the partner communicating stress interpreted it as the partner was not coping with him/her and therefore perceived lower quality of interaction. It could also be the case that the partner expressing their stress did not feel as though they were working together with their partner (Reid et al., 2006).

However, it seems that not only the supporting partner, but the stressed partner also engaged in lower levels of we-talk, as indicated by the negative actor association between one partner’s general stress communication and his/her own we-talk. This is not unexpected given the nature of the couples’ discussions about *external* stress, as external stress traditionally only affects one partner. As such, in this context, it would be appropriate to expect the actor (i.e., the stressed partner) to focus more on his/her own experience of stress. However, as the other partner listens, he/she may choose to emphasize what he/she could do to help alleviate the actor’s stress; thus, they may be more likely to use singular pronouns (e.g., “I,” “you”) than plural pronouns (e.g., “we”).

Previous studies have demonstrated the negative associations between I-talk/you-talk and relationship outcomes and the positive associations between we-talk and relationship outcomes (e.g., Rentscher et al., 2015), especially when partners discuss common stressors, such as coping with cancer (Robbins et al., 2013). This research suggests that there may be instances in which the use of “we” can be more conducive to partners’ perception of we-ness and joint coping efforts than others, such as in activities in which partners participate together (Aron et al., 2000). Further, Slatcher et al. (2008) suggested that the use of “we” in problem-solving discussions is unrelated to relationship quality whereas the use of “we” when describing the relationship or the future of the couple may be linked to relationship quality. These results may be used to educate couples on the importance of viewing external stress as a mutual experience or issue, which is a focus in the Couples Coping Enhancement Program (CCET; Bodenmann and Shantinath, 2004). In addition, the current findings are helpful in establishing considerations for context (e.g., the topic of discussion) and language use in future studies.

Results also showed, as expected, that one partner’s general stress communication was negatively associated with the other partner’s I-talk, which was in turn negatively associated with the first partner’s interaction quality. This finding is consistent with the nature of the external stress conversation that the partner would engage less I-talk when attempting cope with his/her partner because the focus “should” be on their partner’s stress. This finding also supports the extant literature, which shows that I-talk is negatively associated with relationship outcomes (Slatcher et al., 2008: Rentscher et al., 2013).

In addition, one partner’s stress communication was found to be positively associated with the other partner’s you-talk, which was not predictive of the first partner’s interaction quality. This does not support our hypothesis regarding the partner-partner mediation path; however, it was interesting that we found a positive association between the actor’s stress communication and the partner’s you-talk. Again, individuals who engage in positive DC would be expected to use less I-talk because their focus would be directed toward their partners in order to address their partners’ stress; thus, they may be using more “you” words. For instance, in attempting to cope with one’s partner, one may use language like, “Have you tried doing this?” or “You must be feeling so stressed.” You-talk can at times communicate blame and criticism (Biesen et al., 2015) and other times be indicative of support provision, and each use of “you” could have different effects on interaction quality. Thus, the absence of significant associations between the partner’s you-talk and the actor’s interaction quality may be explained by this flexible use of second-person pronouns.

### Emotion-Focused Stress Communication, Emotion Words, and Interaction Quality

Contrary to our hypotheses, we did not find significant mediation for positive or negative emotion words in the association between emotion-focused stress communication and interaction quality. Findings may indicate that the use of positive emotions may be ambiguous. When the actor communicates the negative emotional effects of a stressor and the partner uses many positive emotion words, the actor may perceive that as less supportive than intended rather than supportive. The emotional burden on the actor may prevent him/her from recognizing the positive effects of the partner’s use of positive emotion words and thus may feel as if the partner does not truly understand their negative emotions and is trying to avoid talking about the issue. On the other hand, what we had anticipated to be negative associations turned out to be positive associations between negative emotion words and interaction quality, possibly because the partner’s use of negative emotion words may communicate validation and empathy instead. Again, when assessing language use, it is important to consider the context in which words are spoken (Tausczik and Pennebaker, 2010).

### Problem-Focused Stress Communication, Cognition Words, and Interaction Quality

Despite the support for the mediational effect of cognitive processing words on the association between problem-focused stress communication and interaction quality, the association between stress communication and use of cognition words was negative. That is, when the actor communicated stress by taking a solution-focused approach, the partner used fewer cognitive processing words, which suggested that the partner might not have been engaging in problem-focused dyadic coping. This is similar to the unexpected mediational direction for the use of we-talk, in that stress communication indirectly negatively predicted interaction quality because the partner did not communicate support that the actor may have hoped for. This pattern may resemble demand-withdraw communication styles, in which one partner seeks discussion or resolution of an issue while the other withdraws from the interaction (Christensen and Heavey, 1990). Demand-withdraw communication patterns in couples have been found to be positively associated with relationship distress and dissolution (e.g., Eldridge and Christensen, 2002); thus, our findings appear to be consistent with extant literature.

In summary, results from this study have strong implications in understanding the stress communication and coping patterns of romantic partners. We had originally formulated our hypotheses based on the assumption that the partners in our sample would be effective at dyadic coping; however, some of our findings (i.e., the negative associations between stress communication and we-talk and cognitive processing words) suggested otherwise. The naturalistic design of this study (i.e., no assignment of speaker-listener roles) was integral because it may reveal how couples interact in reality as they switch speaker and listener roles during a stress conversation. The findings from this study could augment existing prevention programs designed to teach couples how to communicate during stress (e.g., CCET; Bodenmann and Shantinath, 2004).

### Limitations

This study is not withstanding limitations. First, there may have been sample bias due to the majority of participants identifying as White and highly educated (i.e., most received at least a college degree). Membership in higher socioeconomic status may affect the way partners communicate, as well as their expectations of effective interactions (e.g., Amato and Previti, 2003). In addition, this study recruited from a population of self-selecting, heterosexual couples. Both partners had to agree to participate, so it was likely that partners were at least moderately satisfied with their relationships in order to complete a research study together. Overall, the lack of representation with respect to this sample may affect how generalizable the results are to all romantic couples facing external stress. For instance, couples who are not native to the United States may encounter immigration stress (Falconier et al., 2013; Falconier et al., 2016) and same-sex couples may experience stress due to discrimination from a heteronormative environment (Randall et al., 2016; Totenhagen et al., 2017). In addition, culture may play a role in how partners communicate and cope with stress (e.g., McCubbin and McCubbin, 1988).

Another limitation of this study was related to the study variables. First, because of the number of existing parameters in our models and the final sample size, we did not include any control variables to ensure sufficient power of our analyses. Possible variables to account for include levels of stress and relationship satisfaction prior to the conversations, because they may impact how couples engage in coping processes together as well as their perceptions of the interactions. Also, this study used observed stress communication as the independent variable, as it has been shown to be associated with various aspects of relationship well-being (Falconier et al., 2015). By having trained raters code for observable behavior, it could reflect a more objective assessment of real-time dyadic coping responses of partners. Further, given that the behavioral coding and linguistic data were aggregated throughout the conversations, these scores may not be representative of the transactional nature of dyadic coping. Specifically, couples engage in discussions about stress, moment-to-moment conversational cues could occur and cause the partners to respond to each other (Mohr and Spekman, 1994; Sanders et al., 1999); thus, using cumulative variables rather than examining the variables at each time point may remove some of the meaningful information about the stress and coping processes during real-time conversations.

Another limitation to the current study is the indistinguishability of partners’ roles. Partners were shown to be indistinguishable based on gender possibly because the assignment of speaker-listener roles was counterbalanced. Thus, an approximately equal number of females and males were expected to be the stress communicator and listener. Speaker-listener roles were also not explicitly assigned in our laboratory instruction, due to an attempt to preserve the naturalistic design of the study. Despite this attempt, it could be that some couples discussed areas of external stress that were salient for both partners. This may explain why speaker-listener roles were not distinguishable in our analyses. Indistinguishability could be an issue when determining the context in which the partners engaged in their word use (e.g., stress communication vs. dyadic coping). While this was one of the notable limitations of the naturalistic design, this design allowed us to examine how couples interact with one another in real-time.

### Future Directions

Future research examining temporal stress communication and coping dynamics may wish to recruit a more diverse sample in terms of ethnicity, education background, and sexual orientation. Doing so could shed light on the possible variability in partners’ stress levels, dyadic coping, language use, interaction quality, and overall relationship outcomes. Generally speaking, having greater variability in these measures would not only increase external validity, but it will allow for a more in-depth knowledge about how stress and coping processes occur for couples. In addition, it may be interesting to consider couples’ conversations about other types of stress in terms of origin (external vs. internal), intensity (major vs. minor), and duration (acute vs. chronic; Randall and Bodenmann, 2009, 2017). Due to their differential impact on the relationship, it is possible that the coping and language processes may be different when discussing different stressors.

One of the advantages of using real-time data is the ability to assess moment-to-moment changes in affect, behavior, and cognition (Laurenceau and Bolger, 2005; Iida et al., 2012). Thus, it is important for future research to collect data and conduct analyses that allow adequate tests of these moment-to-moment fluctuations. Based on this study’s results, we offer several important directions for future research. First, it may be helpful to separate the dialog by speaker-listener turns rather than using the fixed 10-s intervals established by the dyadic coping behavioral coding system (Bodenmann, 2008; Randall et al., 2016). For example, Table 3 displays an example of how data may be organized to facilitate further examination of partners’ stress, coping, and micro-communication processes. Additionally, future research could also utilize statistical analyses that are appropriate in testing for fluctuations between various time points, such as the cross-lagged model (Kenny et al., 2006). The cross-lagged model could be used to examine associations between one partner’s predictor variable (in the case of the present research, stress communication) at Time 1 and the other partner’s outcome variable at Time 2 (i.e., language use). This analytical procedure could be especially helpful in the context of stress communication and coping process because of its transactional nature; further, it would allow researchers to more closely examine the micro-communication dynamics that occur between partners.

**Table 3:** Sample dataset for future studies.

Couple ID	Begin	Gender	S/L	Stress_Begin	Dialog	End	Stress_End	SC	DC
5	0:00:00	Male		50	Man umm so work start with you or me I wonder if that was for both of us	0:00:07	50	88	88
5	0:00:07	Female		50	I’m not sure which one of us it’s supposed to be for we can start with me I guess	0:00:16	50	88	88
5	0:00:16	Male	S	50	I was going to start with rrlike I wonder what kind of questions I should do for this interview today	0:00:20	50	88	88
5	0:00:20	Female	L	50	We should talk about that because it’s probably useful to talk about	0:00:23	23	88	88
5	0:00:22	Male	S	45	I was thinking about the only thing I can think of for now since I’ve already kind of interviewed with them was rrlike uhh rrlike what’s the day to day for I think it’s a processing assistant	0:00:40	23	2	88
5	0:00:40	Female	L	23	So was it rrlike a different position that she interviewed with for	0:00:43	23	88	2
5	0:00:43	Male	S	23	Yes the last one was something else but this one is more like what Will is doing	0:00:48	23	2	88
5	0:00:48	Female	L	23	But I mean obviously you don’t ask him about his job so that would be the question	0:01:03	70	88	1
5	0:01:03	Male	S	70	You typically want to leave work at work unless it’s rrlike something you need to vent about	0:01:06	70	2	88
5	0:01:07	Female	L	70	That’s true umm yeah I don’t know yeah it’s supposed to be rrlike you want to do a really good question but	0:01:26	51	88	2
5	0:01:24	Male	S	57	I don’t know because I don’t necessarily plan on being there for the next 5 years but I don’t necessarily don’t either	0:01:34	63	1	88
5	0:01:34	Female	L	63	Well you don’t know maybe you’re going to super love it and it’s going to be the best thing ever	0:01:43	39	88	2
5	0:01:43	Male	S	39	Do projects at home where it’ll be like I wanted and then just worry about the income and we’re good who knows	0:01:49	39	88	88
5	0:01:49	Female	L	39	You should be honest rrlike you know it’s not like I went to college for loan processing or whatever but see you’re already doing good	0:02:01	50	88	1

*Begin, when the turn begins; S/L, speaker/listener role; Stress_Begin, partners’ stress rating at the start of the turn; End, the time at which the speaking turn ends; Stress_End, partners’ stress rating at the end of the turn; SC, behavioral coding for stress communication; DC, behavioral coding for dyadic coping.*

## Conclusion

The findings from this study suggest language use may mediate the associations between stress communication and perceived interaction quality during real-time interactions. Relationship scholars are encouraged to further explore the interplay between couples’ stress and coping using ecological momentary assessment methods (Kirchner and Shiffman, 2016). Additionally, mental health practitioners working with couples could benefit from implementing psychoeducation or skills training on language use during conversations about stress. Taken together, the way we express ourselves to our romantic partner during stressful interactions can have meaningful effects on how our partner perceives our stress, and how we perceive the interaction and our relationship.

## Ethics Statement

This study was carried out in accordance with the recommendations of the Institutional Review Board at Arizona State University with written informed consent from all subjects. All subjects gave written informed consent in accordance with the Declaration of Helsinki. The protocol was approved by the Arizona State University Institutional Review Board.

## Author Contributions

AR and ND are the PIs on the study (Couples Coregulation Processes during Real-Time Interactions). KL and AR contributed to the conception and design of this study. CT led the collection of the data. KL organized the dataset, performed the statistical analysis, and wrote the first draft of the manuscript. All authors contributed to manuscript revision, read and approved the submitted version.

## Conflict of Interest Statement

The authors declare that the research was conducted in the absence of any commercial or financial relationships that could be construed as a potential conflict of interest.
